# Traumatic Rupture of a Skull Base Dermoid Cyst Mimicking Chronic Meningitis

**DOI:** 10.7759/cureus.25066

**Published:** 2022-05-17

**Authors:** Ahmed Taha, Mahmoud A Abdelrazek, Natalie Manalo, Rabab Elsadek, Scott J Morrin, Alexander Brodski, Aleksandra Augustynowiczd, Roohallah S Mollashahi, Anant Shenoy

**Affiliations:** 1 Radiology, Washington University School of Medicine, St. Louis, USA; 2 Department of Neurology, Mount Auburn Hospital, Harvard Medical School, Boston, USA; 3 Department of Neurology, Massachusetts General Hospital, Harvard Medical School, Boston, USA; 4 Department of Medicine, Mount Auburn Hospital, Harvard Medical School, Boston, USA

**Keywords:** trauma, tumor, rupture, intracranial, dermoid cyst

## Abstract

Cranial dermoid cysts are rare, embryologic tumors containing fat, hair, and other ectodermal elements. They occur most frequently in the posterior fossa and are typically diagnosed as incidental findings on brain imaging done for an unrelated reason. Traumatic rupture of a previously unidentified intracranial dermoid cyst can mimic symptoms of post-concussion syndrome and should be ruled out with magnetic resonance imaging (MRI). Surgical intervention after traumatic rupture may not result in complete symptom control due to the persistence of dermoid cyst debris in the subarachnoid space. Here, we present the clinical scenario and radiological features of a ruptured dermoid cyst due to trauma, highlighting a rare complication of a classically benign lesion.

## Introduction

Dermoid cystic tumors arise at the time of neural tube closure during the third to the fifth week of embryogenesis. These lesions grow slowly [1]; they present during the second or third decades of life. Dermoid cysts are not true neoplasms and enlarge by desquamation and accumulation of sebaceous secretions by dermal elements [2]. They are cystic masses that have thick walls and are lined by keratinized squamous epithelium with ectodermal elements such as hair, sebaceous and sweat glands, teeth, and nails; they also contain fat in varying proportions. They are marginally more prevalent in males than in females [2]. Intracranial dermoid cysts are usually asymptomatic; when symptoms do occur, they result from the mass effect on the adjacent intracranial structures. Rupture is typically spontaneous, although rupture secondary to closed head trauma has been reported in small numbers in literature. Following rupture, the dissemination of the contents of the cyst leads to aseptic chemical meningitis, which in turn can present with headache, seizures, cerebral ischemia, hemisyndrome, and chronic granulomatous arachnoiditis. Moreover, hydrocephalus can also occur [3]. Here, we present a rare case of traumatic rupture of a skull base dermoid cyst.

## Case presentation

**Figure 1:**
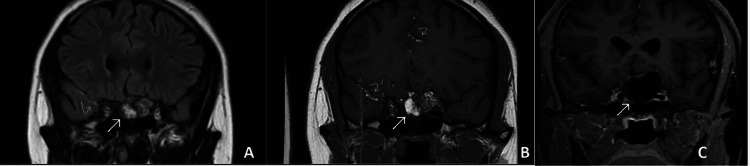
MRI coronal views of the dermoid cyst (straight arrow) and subarachnoid sulcal cholesterol deposits (curved arrows) on FLAIR (A), non-contrast T1 (B), and fat-suppressed non-contrast T1 (C) sequences. Notice the deposits disappear on fat-suppressed sequencing, consistent with their lipid nature.

**Figure 2:**
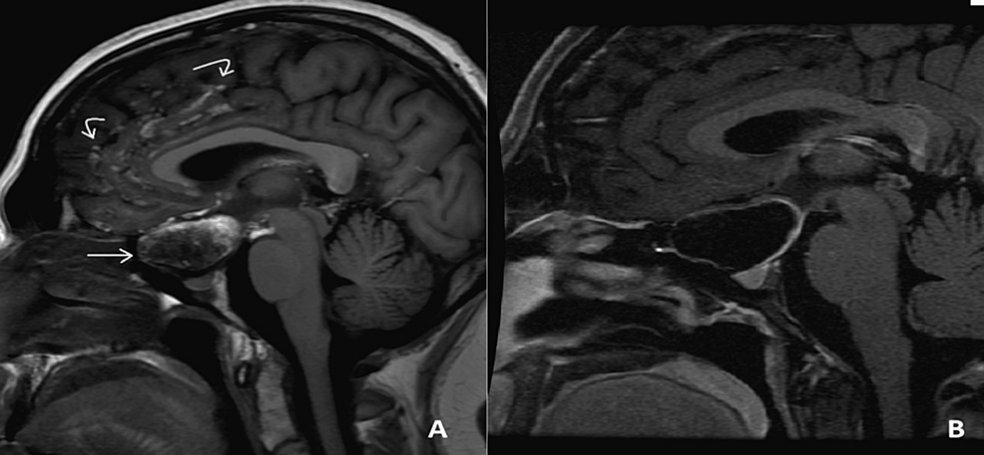
MRI sagittal views of the dermoid cyst (straight arrow) and subarachnoid sulcal cholesterol deposits (curved arrows) on non-contrast T1 (A) and fat-suppressed non-contrast T1 (B) sequences. Again, notice the deposits disappear on fat-suppressed sequencing.

A 24-year-old female with a history of polycystic ovarian syndrome presented with acute onset of headaches, neck stiffness, arm paresthesias, and attention deficits immediately following a whiplash injury sustained during a motor vehicle collision. She was discharged from the ER on the day of the accident with a diagnosis of whiplash and concussion after a normal CT of the cervical spine. Her symptoms continued for two months, for which a magnetic resonance imaging (MRI) of the brain (Figures 1, 2) was performed and showed a heterogeneous cystic lesion in the suprasellar space and punctate T1 hyperintense foci in the subarachnoid space, which disappear on fat-suppressed MRI sequencing, suggestive of lipid particles from a ruptured dermoid cyst. She underwent subtotal transsphenoidal resection of the lesion, with pathological analysis consistent with a dermoid cyst.

**Figure 3:**
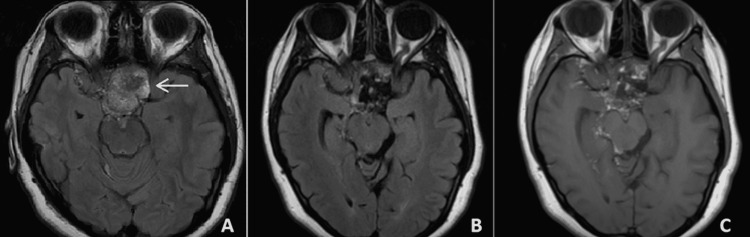
MRI axial views of the dermoid cyst (straight arrow) on preoperative FLAIR (A), postoperative FLAIR (B), and non-contrast T1 (C) sequences.

She continued to have persistent headaches with migrainous features and continued cognitive complaints and paresthesias in the arms bilaterally. Her neurologic examination was normal, aside from attentional deficits. Postoperative imaging (Figure 3B, 3C) showed persistent abnormal signals in the subarachnoid space consistent with lipid particles. Her spinal cord imaging did not show any subarachnoid dermoid cyst components irritating radicular nerves as the etiology of her paresthesias. Although she refused a lumbar puncture, her headaches were attributed to persistent chemical meningitis based on clinical and imaging findings. She was managed symptomatically for headaches and paresthesias with an acceptable response.

## Discussion

Cranial dermoid cysts are rare, and to the best of our knowledge, there are only 10 previously published cases (Table 1) [4-12]. They occur near the midline and arise from epithelial cells abnormally retained during the closure of the neural tube during embryologic development. Unruptured cysts are typically asymptomatic but may, however, present with symptoms of mass effect depending on their location [13]. Although rare, malignant transformation into squamous cell carcinoma has been described [14]. Rarely, cranial dermoid cysts present with spontaneous or (more infrequently) post-traumatic rupture. When dermoid cystic tumors rupture, they spread their contents into the subarachnoid and/or subdural spaces [15].

**Table 1:** Previously reported cases of traumatic rupture of a dermoid cyst. F: female, M: male, VP: ventriculoperitoneal

Author, year	Age	Sex	Cyst location	MRI findings	VP shunting
Phillips et al., 1994 [4]	53	F	Anterior pons	N/A	N/A
Kim et al., 2008 [5]	29	M	Suprasellar	4 cm suprasellar mass with unusual features	No
Park et al., 2012 [6]	28	F	Suprasellar	2.5 cm × 4 cm para- and suprasellar mass, expanding into the left frontal lobe; it induced the midline shifting of 1 cm to the left side	No
Esquenazi et al., 2013 [24]	47	F	Left anterior and middle cranial fossa	A large multi-lobulated lesion extending along the floor of the left anterior and middle cranial fossae	Yes
Skovrlj et al., 2014 [7]	51	M	Anterior to the left temporal lobe	A lesion in the left anterior Sylvian fissure and disseminated foci of subarachnoid fat	No
Ramlakhan et al., 2015 [8]	19	M	Left paracavernous area	Multifocal high signal in a subarachnoid distribution	No
Murrone et al., 2016 [9]	43	F	Left frontal region	A heterogeneously hyperintense irregular lesion (40 mm × 45 mm × 27 mm) with enhancement after gadolinium administration in the left frontal lobe with extension into the left lateral ventricle with hydrocephalus	No
Akbari et al., 2018 [10]	2	M	Left anterior clinoid	1 cm^3^ cystic rim-enhancing lesion adjacent to the left cavernous sinus with an enhancement of the dura of the adjacent anterior temporal pole	No
Zhang et al., 2021 [11]	59	M	Posterior fossa	Lesion located below the cerebellar vermis, with the anterior part extending into the fourth ventricle	No
Das et al., 2022 [12]	49	F	Right frontal lobe	Mildly enhancing fatty and calcific lobulated mass measuring 40 mm × 5 mm in the right frontal lobe extending into the left frontal lobe and along the interhemispheric fissure of the frontal lobe with mild perilesional edema	No

The most common symptoms after the rupture of a cranial dermoid cyst are headache (32%), seizure (30%), transient motor or sensory deficits (16%), and chemical meningitis (7%) [16]. Hydrocephalus is also a concern, although less common [16,17]. Liu et al. found in 2008 that the most common presentations were headaches (57%) and seizures (42%), followed by hydrocephalus (29%) [18]. Sudden-onset headaches and neck stiffness were also reported [19]. Muçaj et al. found that when it comes to imaging, MRI is superior to and more sensitive than CT. On a CT scan, a dermoid cyst can show mixed densities, and contrast administration rarely leads to enhancement [3]. This variability is due to the different contents of a dermoid cyst, as fat, whether intra-cystic or disseminated, is hypodense, and wall calcifications appear hyperdense [20-22].

According to Muçaj et al., it is crucial to understand that dermoid cysts can be easily confused with epidermoid cysts. One difference is that dermoid cysts show fat signals on CT and MRI, whereas epidermoid cysts seem as if filled with CSF [3]. When a dermoid cyst ruptures and spreads, its contents into the surrounding areas and scattered fat droplets, which are hypodense on CT or T1 hyperintense on MRI, may be found floating within the ventricular system and/or the subarachnoid space [3]. Dermoid cysts are only treated when they become symptomatic due to mass effect or when they rupture; the mainstay of treatment is surgical removal and complete excision. Although rare, recurrence may occur [23].

Since the capsule of the cyst may be strongly adherent to a surrounding neurovascular structure, complete removal may not be optimal when assessing the risk/benefit ratio for the procedure. Moreover, disseminated fat droplets are not always easy to remove, which raises the question of whether their presence, which may last for years after the time of rupture, is problematic. According to a literature review published in 2013, intravenous glucocorticoid administration may have a significant role in alleviating symptoms resulting from chemical meningitis [24].

## Conclusions

In the case presented here, we cannot definitively link the motor vehicle collision to the cyst rupture, but our clinical judgment and her presentation suggest this causality. MRI of the brain is the imaging modality of choice for diagnosis as debris from a ruptured cyst can be missed on CT, as was the situation in our patient. The mainstay of treatment for symptomatic cysts suggested by the literature is surgical resection if the location and size are amenable to this option. This may not always result in complete symptom relief due to the persistence of lipid particles and other dermoid cyst debris in the subarachnoid space, contributing to its irritation and continued chemical meningitis for which glucocorticoid therapy may be beneficial.
